# Treatment of Polymeric Films Used for Printed Electronic Circuits Using Ambient Air DBD Non-Thermal Plasma

**DOI:** 10.3390/ma15051919

**Published:** 2022-03-04

**Authors:** Dragos Astanei, Radu Burlica, Daniel-Eusebiu Cretu, Marius Olariu, Iuliana Stoica, Oana Beniuga

**Affiliations:** 1 Faculty of Electrical Engineering, “Gheorghe Asachi” Technical University of Iasi, 700050 Iaşi, Romania; dragos.astanei@tuiasi.ro (D.A.); daniel-eusebiu.cretu@academic.tuiasi.ro (D.-E.C.); marius.olariu@prosupport-consulting.ro (M.O.); oana.beniuga@tuiasi.ro (O.B.); 2 S.C. Prosupport Consulting S.R.L., 707410 Valea Lupului, Romania; 3 Department of Physical Chemistry of Polymers, Petru Poni Institute of Macromolecular Chemistry, 700487 Iaşi, Romania; stoica_iuliana@icmpp.ro

**Keywords:** air DBD plasma, polymer films treatment, adhesion, surface wettability

## Abstract

This study aims to present the properties of the polymeric films after being subjected to DBD plasma treatment in atmospheric conditions. Three different commercial films of polyester (Xerox Inkjet transparencies and Autostat CUS5 Clear film) and polycarbonate (Lexan™ 8010 MC) have been considered for the tests. The surface wettability has been evaluated based on static water contact angle (WCA) for different treatment times varying between 0.2 s and 30 s, the results revealing a maximum WCA decrease compared to a pristine of up to 50% for Xerox films, 75% for Autostat and 70% for Lexan. The persistence of the hydrophilic effect induced by the plasma treatment has also been verified for up to 72 h of storage after treatment, the results indicating a degradation of the treatment effects starting with the first hours after the treatment. The WCA stabilizes to a value inferior to the one corresponding to pristine in the first 24 h after plasma treatment. The adhesion forces, as well as preliminary surface morphology evaluations have been determined for the considered films using atomic force microscopy (AFM). The adhesion forces are increased together with the prolongation of the plasma treatment application time, varying from initial values of 165 nN, 58 nN and 204 nN to around 390 nN, 160 nN and 375 nN for Xerox, Autostat and Lexan films, respectively, after 5 s of DBD treatment. For the considered materials, the results revealed that the plasma treatment determines morphological changes of the surfaces indicating an increase in surface roughness.

## 1. Introduction

Polymer films have been frequently used in many modern industrial applications owing to their high performance. In the area of printed electronics, polymeric materials such as polycarbonate (PC), polyethylene terephthalate (PET) and other films are used as a substrate material for the manufacture of organic light-emitting diodes, sensor applications and organic solar cells [1,2] due to their outstanding flexibility, transparency, fold resistance, tensile strength and chemical and corrosion resistance, low weight and lower cost [3]. The manufacture of printed flexible electronics often requires changing the surface of the polymer to improve adhesion or wetting [4,5]. In this regard, although low-pressure plasma can be used, it was found that atmospheric plasma treatment is more convenient, relatively simple and economical in terms of technology without any vacuum systems suitable for this task [6,7,8]. By using an atmospheric pressure reactor, microscopic structures could be more easily formed on the film substrates through in-line or roll-to-roll processing [9].

Non-thermal plasma has been considered a climate-friendly, sustainable and reliable treatment technology to change the surface of polymers and has a couple of benefits as compared to other methods such as the chemical technique, the electrochemical process or the flame treatment [10]. It can be used to avoid unwanted damage to plasma exposed material in an economical, quick and efficient way. The term “plasma” refers to a partially or fully ionized gas composed essentially of free photons, ions and electrons, as well as atoms in their ground or excited states having a neutral charge. These excited and ionized particles carry enough energy to induce chemical reactions at the interface with solid surfaces that change the properties of materials [11]. In this sense, corona discharge is widely used and has been proven effective in modifying the surface properties of materials but sometimes it has the disadvantage of generating plasma over a small area and the treatment performed can be inhomogeneous [12]. To avoid technical complications that may occur, a dielectric barrier discharge (DBD) is used [13]. A DBD reactor has several advantages such as the flexibility of the design and parameters used, the simple setup, low costs and safety in operation [14].

The DBD reactor has proven its ability to modify different substrates such as metal materials [15,16], wood or MDF boards [17,18,19], various polymeric materials used in the textile, food or electronics industries [20,21,22,23]. Many studies conducted in recent years have shown that treatment by means of an air DBD reactor at atmospheric pressure improves the properties of hydrophilicity, increases the surface tension of polymers, also changing the roughness of their substrates, and has the advantage of uniform and fast treatment of the entire surface [24,25,26,27].

A different group of authors assessed the influence of plasma treatment on different types of substrates and materials as reported below. Van Dogen et al. in [1] studied the influence of plasma treatment on polyethylene naphthalate (PEN), polyethylene terephthalate (PET), polycarbonate (PC), fluorinated ethylene propylene (FEP) and polyimide (PI) polymer films and highlighted increased wetting behavior after the treatment due to an increase in the polar part of the surface energy. Azimet et al. in [20] studied the influence of DBD plasma treatment on polypropylene (PP) films and showed that the treatment conducted to a decrease in the contact angle while the surface roughness increased with the treatment time. AFM and diffuse reflectance spectroscopy analysis showed a remarkable increase in hydrophilicity. Leroux et al. in [24] treated a polypropylene film using DBD plasma and observed increased surface free energy and nano-structural changes of the polymer film as bumps of oxidized polypropylene. Moreover, XPS analyses presents an increase in the polypropylene film surface oxygen contents. Kelar et al. in [28] assessed the plasma treatment effect on polycarbonate surfaces and emphasized changes in wettability for the treated surfaces in terms of WCA and surface free energy. Sikora et al. in [29] applied plasma discharge on poly(methyl methacrylate) (PMMA) polymer recording a water contact angle decrease from 83° to 29.7° and improved wettability. Niu et al. in [30] exposed polyethylene terephthalate (PET) and polytetrafluoroethylene (PTFE) under DBD discharge and reported relevant WCA variation for both polymers and improved hydrophilicity of the surfaces.

PET film has been used in a wide range of applications (packaging, printing, capacitors) because it has some good properties and is easy to recycle and it has a comparatively lower cost which made it very popular despite its inadequate wettability and adhesion properties [31]. Additionally, PC has high creep resistance, durability, high hardness and excellent optical transparency, being an attractive polymer. PC is used in a variety of applications such as a substitute for glass, plastic vessels, in agriculture, constructions, and commercial design. However, the adhesive strength between the deposited film and the PC substrate is weak due to the low surface energy. Therefore, in order to increase the surface tension, it should be activated by DBD non-thermal plasma treatment [29].

The main idea of this study is to present the results obtained for three commercially available polymeric substrate surfaces: Xerox inkjet, Lexan and Autostat foils, which have been exposed to an air DBD plasma at atmospheric pressure with the aim of modifying the water contact angle (WCA), the adhesion force and the surface morphology changes.

The polymeric substrates assessed in this paper are commonly used for printing technologies and their study aimed at a possible improvement of the quality of the material for certain printing technologies. The goal is to compare the effect of DBD plasma on different types of materials (Lexan—polycarbonate, Xerox and Autostat—polyester films) as well as different types of foils manufactured from similar material—Xerox and Autostat. Autostat foil is a dedicated substrate for electronic circuits printing while Xerox foil is a substrate predominantly used for inkjet common printing.

As the non-thermal plasma treatment effect of the considered film surfaces is not constant over time and there are differences depending on the treatment time, it is important to establish how wettability changes with the treatment time, the storage time and the oxidation ageing of these treatments.

Industrial plasma treatments involve continuous and rapid treatment surfaces of large quantities of samples. The diminution of treatment time of such individual samples by a few milliseconds can finally result in the reduction in treatment time of thousands of samples by a few minutes. The primary objective of this study is to determine the optimal treatment time of a polymer surface, which is thus very important for industry.

The results and treatment method presented in this paper can serve as a background for applying the air DBD plasma treatment in order to increase the hydrophilicity of the polymeric films used for flexible printed electronic circuits. The paper focuses on the use of plasma-based technology for pretreating the surface of the polymeric substrate at atmospheric pressure and in the absence of additional gases within the printing manufacturing process (both screen and inkjet printing). The DBD plasma represent a technological solution which provides high energy input at a molecular level, at room temperature and atmospheric pressure, achieving an important interface between the target and the discharge, as well as the polarization of the occurring reactive species.

## 2. Materials and Methods

For this experiment, three different types of commercial polymeric films have been considered. The first polyester substrate tested was Xerox Inkjet transparencies (003R98197) films having a wide compatibility with a large number of inkjet printers and a lower cost. Experiments have also considered a polycarbonate film—Lexan™ 8010 MC (polycarbonate). It has a polished surface and high clarity, being usually used in applications relating to electronics, automotive interiors or general electrical appliances. The polymeric foil Autostat film (polyester film) of the type CUS5 Clear, (untreated, 125 micron) has been also treated. Autostat is used for the manufacture of membrane and conventional switch circuitry layers such as computer keyboards.

**Figure 1:**
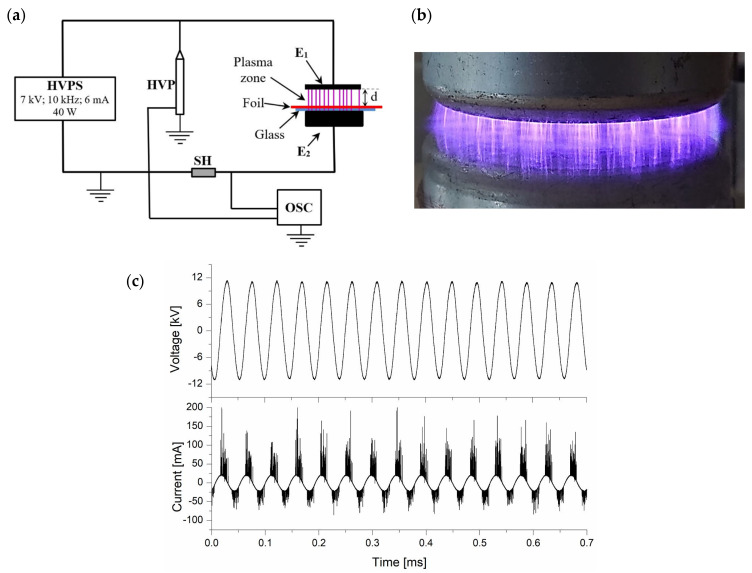
DBD reactor (**a**) schematic diagram; (**b**) DBD discharge streams; (**c**) current and voltage waveforms.

The dielectric barrier discharges (DBD) were generated in atmospheric air using a reactor with a dielectric between the two circular 60 mm diameter plane electrodes, E_1_ and E_2_, respectively, which is covered by a glass plate forming a 3 mm thick dielectric barrier. The distance between the electrodes is 7 mm. The schematic diagram and the image of the non-thermal plasma discharge are shown in Figure 1a,b, respectively. Non-thermal plasma is generated using a high voltage power supply (HVPS) which generates about 20 kHz, an output voltage of 7 kV, the average power per discharge being 40 W, for a 6 mA current.

The polymer layer is placed at a 4 mm distance from E_1_ electrode. When the voltage applied exceeds the electrical breakdown threshold, an avalanche ionization phenomenon initiates. Due to this phenomenon, a multitude of plasma streamers occur between the electrode and the dielectric glass. The discharge stops when the voltage passes to zero and re-initiates when the voltage changes the polarity.

The use of the dielectric barrier prohibits the evolution of the electric discharge into electric arc. At the same time, the charge carriers accumulated at the dielectric surface, quenches the discharge by lowering the field strength. In the discharge, there are many streams exhibiting concurrently and individually, with a lifespan in the range of nanoseconds.

In order to assess the discharge current, it was used a TT-HVP-2739 high voltage probe (HVP) with a divider ratio of 1000:1, a 100 Ω resistor (SH) and a LeCroy 454 oscilloscope to record the waveforms presented in Figure 1c.

Tests were focused on commercial PET foils (Xerox transparency foil used for laser and color laser printers and Autostat film used for flexible circuits with conductive silver inks) and on a polycarbonate film (Lexan). The thickness of the polymerc surfaces were around 100–125 µm.

**Figure 2:**
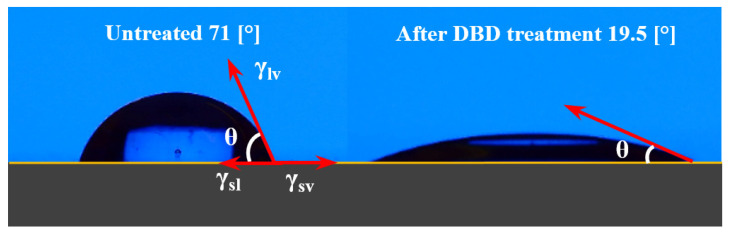
The WCA for the treated Autostat foil decreased from 71.2° down to 19.5°.

Sessile drop method is a common technique used for flat surfaces allowing water contact angles to be measured from the drop profile. In the present paper, the authors used an Ossila Contact Angle Goniometer to assess the WCA and the surface free energy of liquid droplets. A calibrated distilled water drop is settled on the polymer surface using a 25 µL syringe provided with a blunt needle (Φ = 0.47 mm). Figure 2 shows the WCA after 30 s of DBD treatment for the Autostat foil, highlighting hydrophilic enhanced properties.

There are many factors affecting the WCA including impurities, porosity, surface roughness, or surface free energy. The correlation between the water surface tension and the substrate surface energy is described by Young’s Equation (1) where γ_sv_, γ_sl_, γ_lv_ and θ are the interfacial tensions of the solid-liquid, solid–vapor, liquid-vapor interfaces and the Young’s contact angle, respectively [32]:

(1) $$\gamma_{\mathrm{sv}}=\gamma_{\mathrm{sl}}+\gamma_{\mathrm{lv}}\cdot \cos\theta$$

According to Equation (1), when the contact angle is less than 90° the surface is hydrophilic while for a contact angle higher than 90° the surface is hydrophobic.

The morphology and the local mechanical properties at the nanoscale were investigated by means of atomic force microscopy (AFM) in semi-contact and in contact mode, respectively, in atmospheric conditions, at room temperature, on surface areas of 5 × 5 μm^2^, using a NTEGRA multifunctional Scanning Probe Microscope (NT-MDT Spectrum Instruments, Zelenograd, Moscow, Russia) with NSG10 high-resolution AFM probes (NT-MDT Spectrum Instruments, Zelenograd, Moscow, Russia). The free resonant frequencies of the cantilevers were 281 and 336 kHz, and the corresponding cantilever’s normal spring constants were 10.5 and 20.1 N/m. The AFM data acquisition and analysis were performed using Nova 1.1.1.19891 software from NT-DMT. The average values of the adhesion force, F_adh_, were calculated using force-distance spectroscopy in contact mode, starting from the retract force–distance curves, namely DFL (height) curves, according to Hooke’s law:

F_adh_ = −k∙Δx (2)

where k is the cantilever stiffness and Δx is the deflection of the cantilever in relation to the sample. The abbreviation DFL comes from the normal deflection distribution of the cantilever. For each type of polymeric film tested, two samples have been treated and analyzed, the results being compared with the control. For each sample, up to 10 scanning areas were considered to determine the adhesion forces and to evaluate the surface morphology.

The adhesion forces were measured one hour after the plasma treatment. The surface morphology was evaluated several days after the treatment.

The experimental set up used 3 types of substrates—polymeric films, and the goal of the study was to evaluation the air DBD treatment effect in order to enhance the printing capabilities on these surfaces used in printable flexible electronics.

## 3. Results

### 3.1. Water Contact Angle (WCA) Evolution

The time evolution of the water contact angle (WCA) on the polymeric surface, for different plasma treatment time spans, has been studied for three different materials commonly used for electronic circuits printing: Xerox foils, Autostat foils (PET) and Lexan (polycarbonate).

Contact angle measurement is a relevant method to identify the hydrophilicity of polymeric surfaces. The adhesion of a surface is related to its wetting. To assess the surface wetting, contact angle measurement may be performed for a liquid droplet in contact with a solid surface.

**Figure 3:**
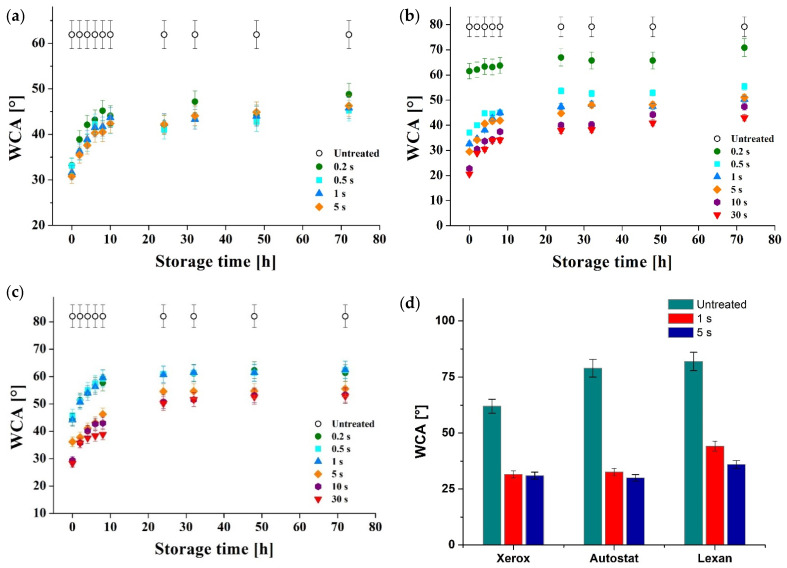
WCA evolution in time after the DBD treatment application: (**a**) Xerox inkjet transparency foil; (**b**) Autostat film (polyester film—PET); (**c**) Lexan foil (polycarbonate); (**d**) WCA evolution after 1 s and 5 s of DBD treatment for Xerox, Autostat and Lexan films.

Figure 3 illustrates the WCA evolution in time after the DBD treatment application for the considered substrates (a) Xerox, (b) Autostat and (c) Lexan, and a comparison between the immediate effect of plasma treatment on the water contact angle in the case of the tested substrates, (d).

It is shown that in the case of Xerox foils—Figure 3a—even after a very short time of exposure of the foil to the DBD reactor of several hundred milliseconds, the hydrophilic properties increase, and the WCA decreases from 60° to around 30° for 200 ms of treatment. There are no important differences concerning the WCA values obtained after the plasma treatment between the different treatment times considered (from 0.2 up to 5 s). The WCA measurements indicate an important increase of contact angle over time, just after DBD treatment. This aspect implies that the treated foil must be used immediately after treatment for the best physical wetting property. After 24 h of storage from plasma application, the WCA values increase up to 45° while remaining relatively stable even for more than 70 h of storage. In the case of the Xerox type polymeric substrate, the plasma treatment could not exceed 5 s due to the thermal degradation (deformation) of the foil.

Figure 3b reports the evolution in time of WCA for the Autostat film (PET) from the initial moment after DBD plasma treatment up to 72 h (storage time from the initial non-thermal plasma treatment of the PET surface). It has been shown that for the untreated foil, the initial contact angle is around 80° and that it decreases down to 20° for a DBD treatment applied for 30 s.

Within the first 30 s after subjecting the material to DBD treatment, the values of the WCA are around 20°, highlighting a very good hydrophilicity, while after 72 h after the plasma treatment, the WCA increases to values close to 45°, a fact which indicates that the hydrophilic properties decrease with storage time. As for the DBD treatment performed at 200 ms, the lowest WCA values of approximately 60° are recorded in the first 8 h of ageing, 20° higher than the treatment time of 500 ms which indicates that the properties of the material change rapidly and vary depending on the polymer residence time in the plasma zone.

Figure 3c illustrates the WCA evolution of universal printing transparency foils of the Lexan type. Similar to PET foils, the hydrophilic properties increase after the plasma treatment, the WCA decreasing from 80° to around 45° after 0.2 up to 1 s of treatment. For longer exposure of the film to plasma treatment, of up to 30 s, the WCA reaches lower values, up to 30°. It is also shown that WCA increases over time, but the WCA values stabilize after 24 h of storage.

After 24 h since the plasma treatment has been applied, the WCA increases from 30° to 50° in the case of 30 s of plasma treatment applied on the polymeric surface and from 45° to 60° in the case of the foil treated for 1 s. Regardless of the substrate plasma treatment time, the WCA angle stabilized after 24 h after treatment at a value comprised between 50° and 60°.

As we can see in Figure 3d, the most important effect of the DBD plasma treatment on the contact angle (WCA) of the printing foils was registered by the Autostat film where the contact angle decreased by 50° followed by Lexan foil with a decrease of 45° and the Xerox film with a decrease of 30°. In all cases, after a storage time of 72 h, the contact angle stabilized to around 50°, and did not return to the original value of the untreated foils.

### 3.2. Adhesion Force and Morphology

The AFM analyses emphasize significant modifications of the local mechanical properties at the nanoscale, investigated from the force–distance curve DFL (height) acquisition using force–distance spectroscopy, as well of the surface morphology after the non-thermal plasma treatment. For all the considered polymeric films, the adhesion force increases after treatment, the values of the adhesion force being influenced by the treatment time.

**Figure 4:**
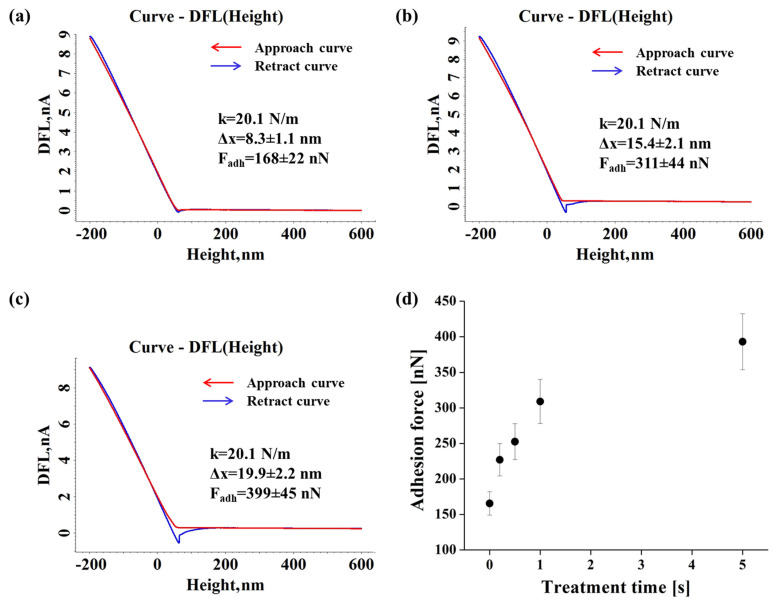
Representative force-distance DFL (height) curves recorded for the pristine (**a**) and DBD plasma treated for 1 s (**b**) and 5 s (**c**) Xerox inkjet film and the adhesion force evolution with the treatment time (**d**).

Figure 4 depicts the representative approach/retract force–distance DFL (height) curves used to calculate the average values of the adhesion force for the untreated and DBD plasma treated for 1 s and 5 s Xerox inkjet film, as well as the adhesion force evolution with the exposure time. The cantilever stiffness (k), the mean deflection of the cantilever in relation to the sample (Δx) and the average value of the adhesion force (F_adh_) are highlighted in each case.

The adhesion force increases from an initial value of around 170 nN, corresponding to the untreated substrate, up to 400 nN for a 5 s treatment. The results emphasize the DBD plasma treatment efficiency and its significant influence on the adhesion force values for all the considered treatment times, even for 0.2 s.

**Figure 5:**
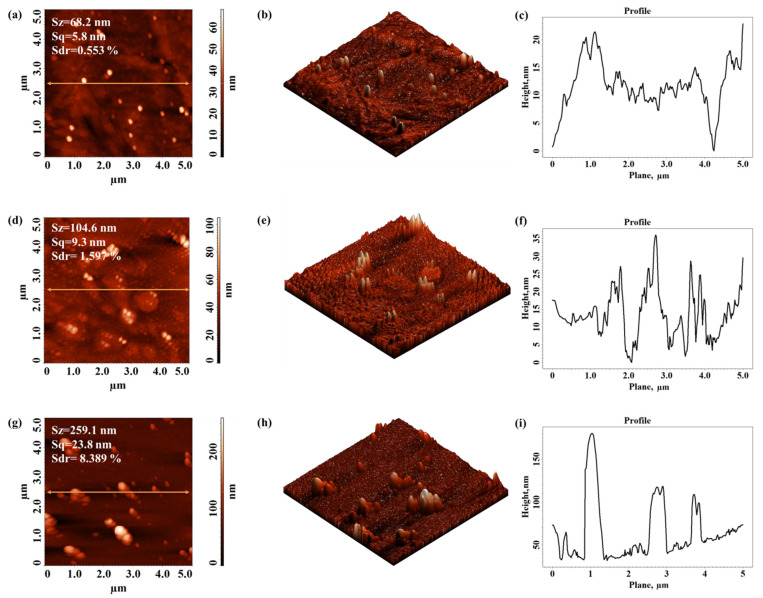
Two-dimensional and three-dimensional topographical AFM images and corresponding cross-section profile taken along the highlighted line obtained for Xerox Inkjet film: not treated (**a**–**c**) and after 1 s (**d**–**f**) and 5 s (**g**–**i**) of DBD plasma treatment.

Figure 5 presents the surface morphology of the Xerox Inkjet film in the initial state (untreated) and after 1 s and 5 s of DBD plasma treatment. The corresponding cross-section profiles taken along the highlighted lines from the topographical images were also presented. Figure 5 emphasizes a series of changes in surface morphology after plasma treatment, evaluated by means of three 3D-texture parameters, namely Sz—the maximum height of the surface, Sq—the root mean square roughness of the surface, and Sdr—the surface area ratio, the last one being used to estimate the complexity of the morphological features. The values of these calculated parameters are shown for every characteristic AFM image. After 5 s of DBD plasma treatment the number of protuberances as well as their heights on the polymer surface increase. The height of the protuberances can reach values of up to 250 nm (Sz) after a 5 s plasma treatment. These surface features lead to a significant increase in the roughness (Sq) for the plasma treated sample by about four times as compared to the one obtained for the pristine sample, and also, an obvious enhancement in the morphology complexity (the value of the Sdr goes up from 0.553% to 8.389%).

The surface morphology reveals the existence of protuberances on the surface that may be due to local heating under the action of plasma streams which affect the surface roughness.

**Figure 6:**
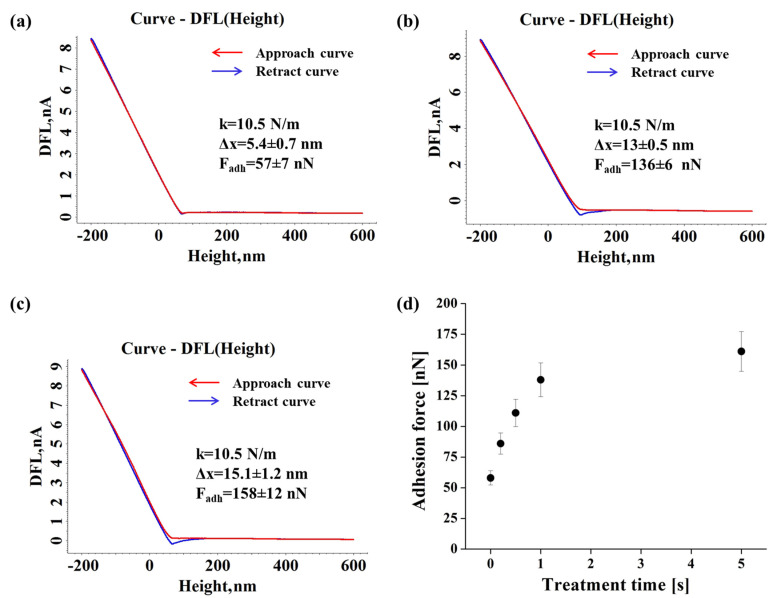
Representative force–distance DFL (height) curves recorded for the pristine (**a**) and DBD plasma treated for 1 s (**b**) and 5 s (**c**) Autostat film and the adhesion force evolution with the treatment time (**d**).

Figure 6 presents the approach/retract deflection curves for Autostat film before and after it was subjected to DBD plasma treatment for 1 s and 5 s, and the adhesion force evolution with the plasma treatment time.

In the case of Autostat film, the force–distance curves emphasize lower values for the adhesion forces as compared to those obtained for the Xerox Inkjet film under the same conditions. The adhesion forces are directly influenced by the treatment time, slightly increasing from around 60 nN (obtained for the untreated surface) up to about 160 nN after 5 s of DBD treatment and tend to stabilize at 200 nN.

**Figure 7:**
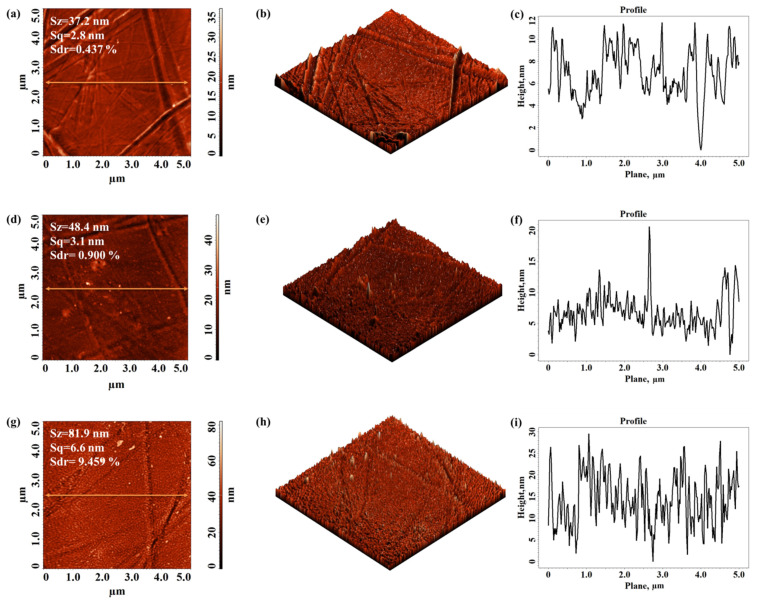
Two-dimensional and three-dimensional topographical AFM images and corresponding cross-section profile taken along the highlighted line obtained for Autostat film: not treated (**a**–**c**) and after 1 s (**d**–**f**) and 5 s (**g**–**i**) of DBD plasma treatment.

Figure 7 presents the initial surface morphology of the untreated Autostat film and after 1 s and 5 s of DBD treatment, respectively, along with the texture roughness parameters and corresponding cross-section profiles.

The Autostat surface has lower roughness parameters as compared to those obtained for the previously analyzed Xerox Inkjet film, before and after plasma treatment. The effect of plasma on the Autostat substrate is more obvious than in the case of Xerox film, affecting the entire analyzed surface, not just some regions of the surface. In this case, the protrusions obtained after treatment are lower in height (see the values of the Sz) but are more numerous, developing an enlarged complexity of morphology (until around 9.5%).

**Figure 8:**
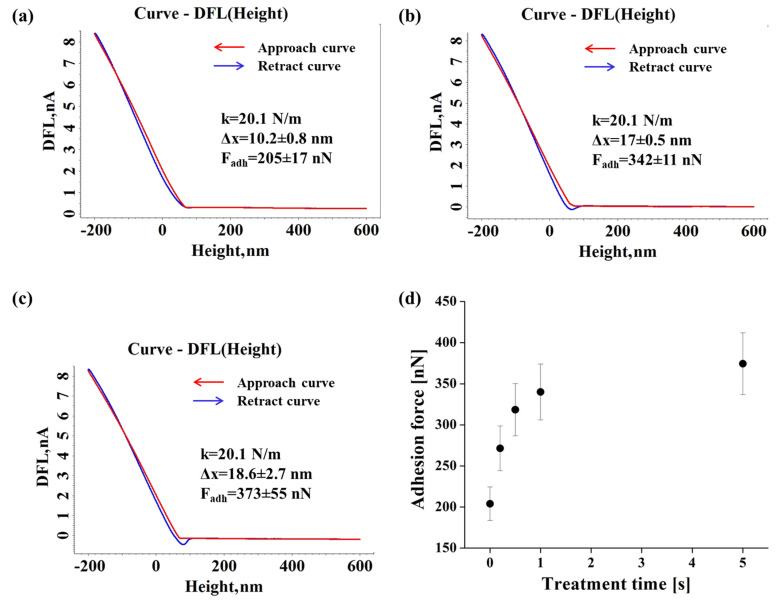
Representative force–distance DFL (height) curves recorded for the pristine (**a**) and DBD plasma treated for 1 s (**b**) and 5 s (**c**) Lexan film and the adhesion force evolution with the treatment time (**d**).

Figure 8 introduces the exponent AFM force–distance DFL (height) curves recorded for the untreated and DBD plasma treated for 1 s and 5 s Lexan film and the evolution of the average adhesion force with the treatment time.

In the case of Lexan film, the DFL curve shows higher differences after 5 s of DBD treatment than in the case of Autostat film, but similar to Xerox Inkjet film. The initial values of the adhesion forces, corresponding to the untreated surface is around 200 nN. The adhesion force tends to stabilize after 5 s of treatment to about 375 nN, within the same range of values as the Xerox film.

**Figure 9:**
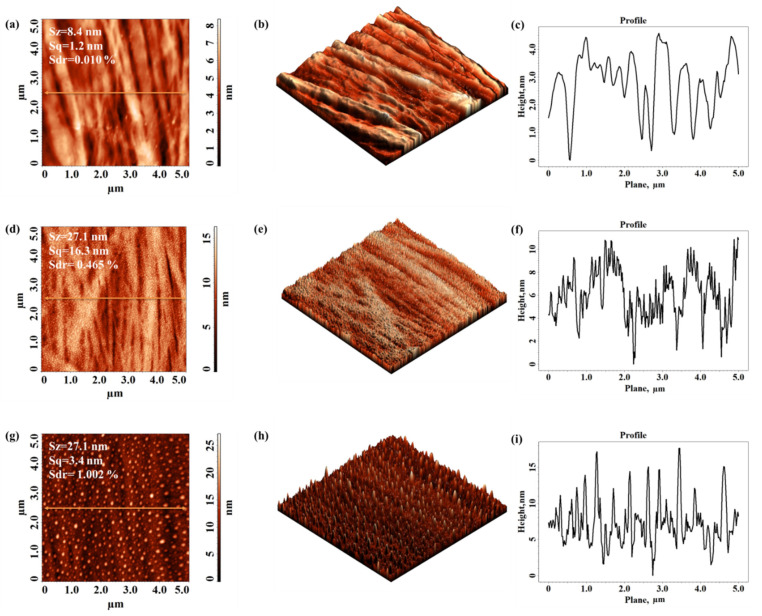
Two-dimensional and three-dimensional topographical AFM images and corresponding cross-section profile taken along the highlighted line obtained for Lexan film: not treated (**a**–**c**) and after 1 s (**d**–**f**) and 5 s (**g**–**i**) of DBD plasma treatment.

Figure 9 shows the initial surface morphology of Lexan film (untreated) and after 1 s and 5 s of DBD treatment, along with the corresponding cross-section profiles. The effect of plasma on the surface appearance of Lexan substrate is more obvious in comparison to previously presented cases. Initially, Lexan film surface was very smooth, with a small roughness (Sq) around 1 nm and small intricacy (Sdr of 0.01%). The well-defined globular formations constituted after the treatment are lower but more numerous and more evenly distributed on the surface than in the case of the Xerox sample. The slightly increasing complexity of the morphology was influenced by these similar bumps which monotonously cover the initial relief.

**Figure 10:**
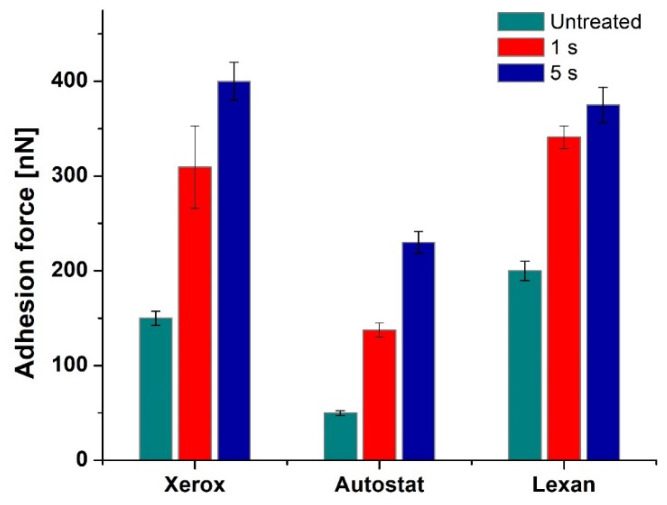
Variation of the adhesion force for Xerox, Autostat and Lexan films after 1 s and 5 s of DBD treatment.

Figure 10 presents a comparison of the adhesion forces after 1 s and 5 s of DBD treatment for all the polymeric films under study, namely: Xerox, Autostat and Lexan.

Figure 10 shows that the most important effect of DBD plasma treatment was registered by the Xerox film where the adhesion force increased by 250 nN, after 5 s of treatment, followed by the other types of printing foils Autostat and Lexane with a similar increase of 170–180 nN.

## 4. Discussion

This paper presents the effect of treatment of three different types of polymer surfaces for printing flexible electronic circuits by cold plasma electrical discharge DBD.

The efficiency of plasma treatment on different materials has been assessed in various studies, such as in [33] treating poly(lactide) (PLA) films by diffuse coplanar surface barrier discharge plasma, in [34] it was treated by oxygen plasma the silicon, silicon dioxide and glass, in [35] it was treated biaxially oriented polypropylene (BOPP) film by corona discharge or [36] epoxy polymer in air DBD and gliding arc, the results showing significant effects of the plasma treatment on polymeric surfaces. The most common surface modifications reported refer to wettability increasing of treated surfaces, surface oxidation, increase of adhesion forces and morphological changes,

Plasma treatment can effectively change the chemistry of the surface layer of polymeric materials, increasing their polar character and the surface energy, thus improving their bondability, as reported in [34]. Surface changes due to plasma treatment represents a process of interaction between the plasma and the treated material. The DBD plasma generates reactive species, which along with the free electrons and the UV radiation can break chemical bonds at the surface leading to surface etching. Moreover, the oxygen containing radicals can determine the generation of polar groups at the surface of the polymer, modifying its chemistry [37,38]. The oxygen-based polar groups enhances the hydrophilicity of the polymer surface, as reported in [30,39].

The non-thermal plasma treatment of polymer surfaces leads to a significant and fast reduction (40–50%) in the water contact angle of the substrate surface after 200–500 ms. The fast decreasing of the WCA (after hundreds of ms) is an important piece of information especially for roll-to-roll applications, when the treatment time of the foils should be very short due to the high speed of the polymeric foils subject to the printing process. After the plasma treatment application, the contact angle follows an increasing trend that stabilizes to a constant value (50–60% of the initial contact angle value of the material) after about 24 h. This value remains constant even after 72 h. This phenomenon can provide important information regarding the evolution of the physical-chemical characteristics of polymeric surfaces treated with non-thermal plasma especially when printing is done after a certain time after treatment or after a certain time of transport and storage of polymeric films.

Furthermore, the influence of non-thermal plasma treatment on the morphology and the evolution of adhesion forces for the three types of substrates, namely Xerox, Autostat and Lexan, was studied. Plasma treatment significantly modifies the morphological characteristics of the polymer surface and simultaneously leads to an increase of the adhesion force on the surface. The experimental data shows that that the most important effect of DBD plasma treatment was on the Xerox film where the adhesion force increased by 250 nN, after 5 s of treatment, followed by the other types of printing foils Lexan and Autostat with an increase of 170–180 nN and 100 nm, respectively.

## 5. Conclusions

The present study emphasizes a significant decrease in WCA of non-thermal plasma treated polymer film surfaces (around 50% from initial WCA) for all film types considered after only 0.2–0.5 s. This aspect is important for the treatment of the foils used in in-line printing systems where the speeds of the foil movement are very high. Another important factor is the stabilization of the WCA 24 h after the treatment with non-thermal plasma, which is a useful piece of information in the case where the printing is performed after a longer time after the treatment (or in another location) or for the storage of the treated foils.

AFM analyses showed an important effect induced by plasma treatment on the polymeric surfaces and an increase of the adhesion forces with the treatment time.

The experimental data obtained highlighted the necessity to adapt the treatment time to each type of material used, in order to obtain the desired parameters.

## Data Availability

The data presented in this study are available on request from the corresponding author.
